# Complete traumatic main pancreatic duct disruption treated endoscopically: a case report

**DOI:** 10.1186/1752-1947-8-173

**Published:** 2014-05-31

**Authors:** Antonios Vezakis, Vasilios Koutoulidis, Georgios Fragulidis, Georgios Polymeneas, Andreas Polydorou

**Affiliations:** 1 2nd Department of Surgery, Aretaieion Hospital, 76 Vas. Sofias Ave., Athens 11528, Greece; 2 Department of Radiology, Aretaieion Hospital, 76 Vas. Sofias Ave., Athens 11528, Greece

**Keywords:** Endoscopic treatment, Pancreatic duct disruption, Pancreatic trauma

## Abstract

**Introduction:**

Pancreatic injury is uncommon and the management remains controversial. The integrity of the main pancreatic duct is considered the most important determinant for prognosis.

**Case presentation:**

A 19-year-old Greek man was referred to our tertiary referral centre due to blunt abdominal trauma and an associated grade III pancreatic injury. He was haemodynamically stable and his initial treatment was conservative. Due to deterioration in his clinical symptomatology he underwent an endoscopy 20 days postinjury, where a stent was placed in the proximal pancreatic duct remnant and a bulging fluid collection of the lesser sac was drained transgastrically. He made an uneventful recovery and remains well 7 months postinjury, but a stricture with upstream dilatation of his main pancreatic duct has developed.

**Conclusions:**

The clinical status of the patient rather than the grade of pancreatic injury should be the principal determinant to guide treatment. Endoscopic stenting and drainage is an attractive minimally invasive procedure and it may obviate the need for surgery. However, further investigation is required regarding the safety and outcome.

## Introduction

Non-operative treatment of abdominal injuries has become the standard of care in haemodynamically stable patients with blunt abdominal trauma due to advances in intensive care management and non-operative treatment options. Pancreatic injury occurs in approximately 5% of patients with blunt abdominal trauma and is associated with a mortality of up to 30% and a morbidity of up to 45%. The integrity of the main pancreatic duct (MPD) is the most important determinant of prognosis in these patients, with disruption to the MPD an indication for laparotomy [1-4].

The present case report describes a patient with complete MPD disruption, who was treated non-operatively by endoscopic means.

## Case presentation

A 19-year-old Greek man was referred to our tertiary referral centre, from a district hospital, due to blunt abdominal trauma with an associated pancreatic injury, 5 days ago. The mechanism of injury was a blow into his epigastrium during an assault.

**Figure 1:**
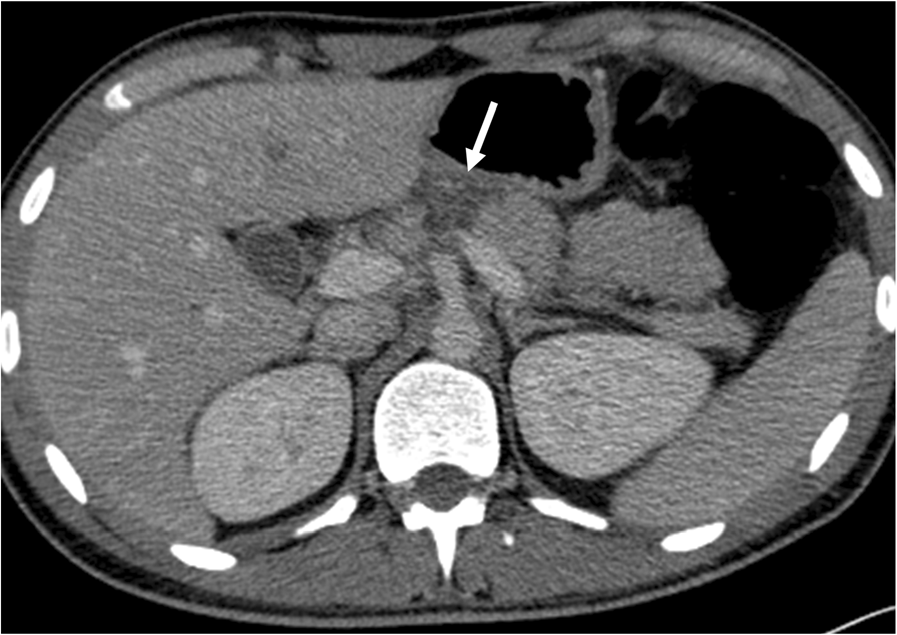
Contrast-enhanced computed tomography of the abdomen reveals a full thickness laceration of the pancreatic neck (arrow) with a lesser sac fluid collection (not shown), suggesting pancreatic duct disruption.

**Figure 2:**
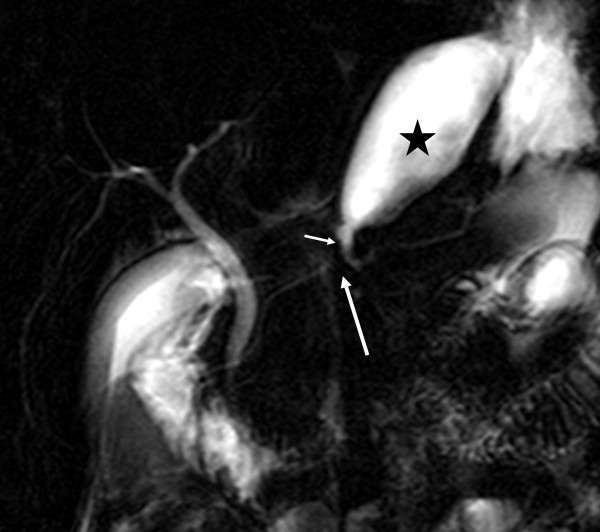
**Magnetic resonance cholangiopancreatography shows complete disruption of the pancreatic duct (long arrow).** A communication (short arrow) between the duct upstream of the disruption and a fluid collection (asterisk) is also clearly demonstrated.

**Figure 3:**
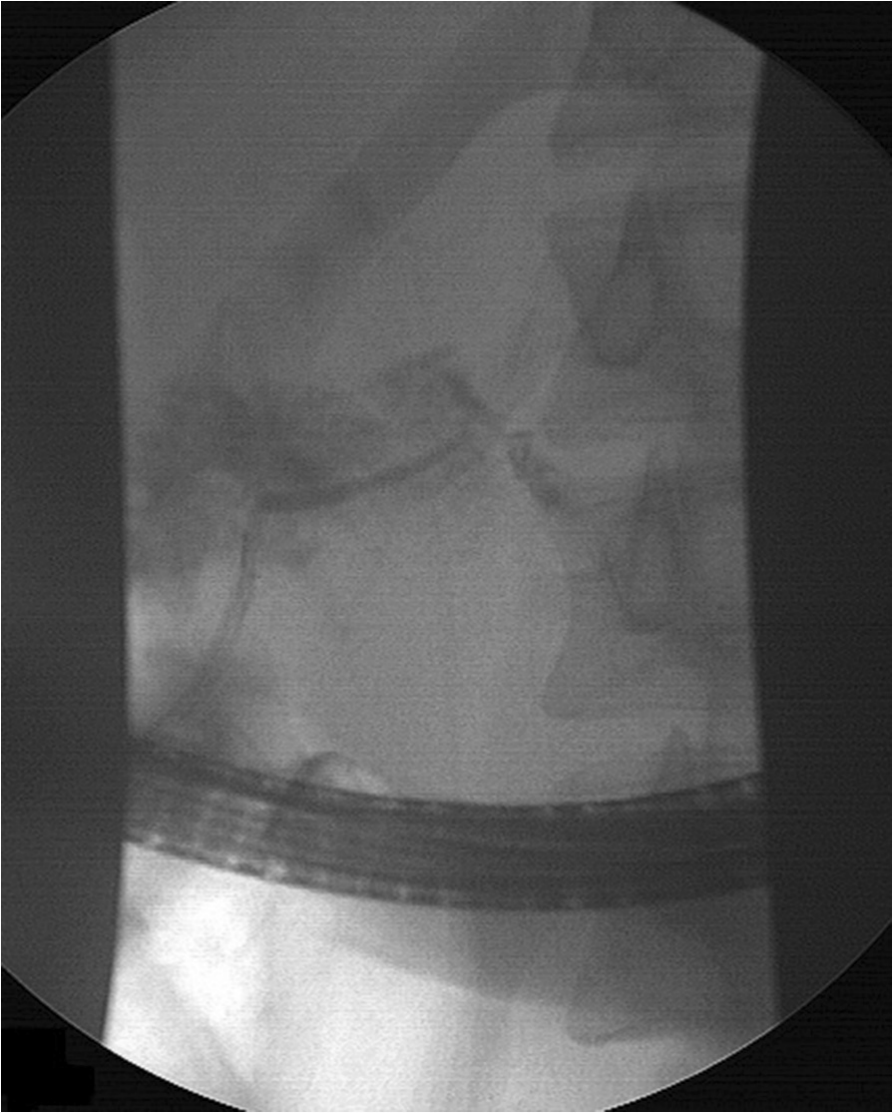
Cannulation from the minor papilla shows complete transection of the main pancreatic duct with extravasation of contrast and no opacification of the distal pancreatic duct.

**Figure 4:**
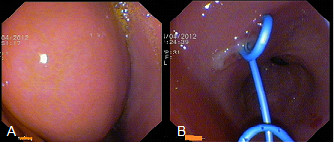
**Endoscopic view. A**. The bulging at the posterior wall of the stomach due to the fluid collection in the lesser sac. **B**. Two double pigtail stents were placed transgastrically to drain the fluid collection.

**Figure 5:**
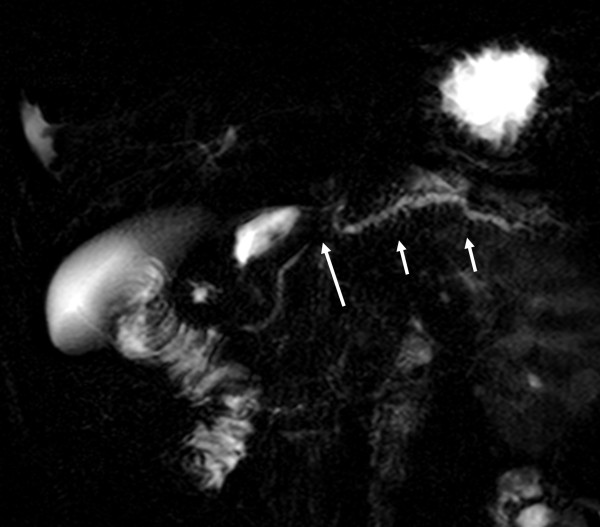
**Follow-up magnetic resonance cholangiopancreatography performed 6 months postinjury.** A stricture suggesting complete disruption has developed (long arrow), with upstream dilatation of the main duct and its side branches (short arrows).

He was haemodynamically stable and clinically there was mild tenderness in his epigastrium without any signs of peritonism . Laboratory tests showed a mild increase in serum amylase (670U/L, reference values 25 to 125) and C-reactive protein (9.1, reference values 0 to 0.5).An abdominal computed tomography (CT) scan documented the injury with a full thickness laceration of his pancreatic neck and an associated 6×2cm fluid collection in this lesser sac with a minimal account of free fluid in the rest of his peritoneal cavity (Figure 1). Magnetic resonance cholangiopancreatography (MRCP) showed a complete MPD disruption at the level of the neck (Figure 2). He was treated conservatively with fasting, total parenteral nutrition and octreotide. During observation and on the 16th postinjury day, he became pyrexial with an increase in his white cell count and amylase level. A repeat CT scan showed enlargement of the fluid collection. Because he remained haemodynamically stable with no signs of peritonism, it was decided to undergo an endoscopic retrograde cholangiopancreatography (ERCP) with possible stenting of the MPD or internal drainage of the fluid collection. The decision was based on persisting fever and enlargement of the fluid collection despite the already mentioned treatment and antibiotics.An ERCP, performed 20 days postinjury, showed a complete pancreas divisum and cannulation from the minor papilla showed a complete MPD transection with extravasation of contrast (Figure 3). The MPD could not be bridged with a guidewire. A pancreatic pigtail stent (Cook Medical) was placed in the proximal MPD to facilitate drainage of the proximal pancreas to his duodenum. The collection of the lesser sac was bulging into the posterior wall of his stomach and a transmural drainage was performed with simultaneous placement of two 7 Fr, 4cm double pigtail stents (Cook Medical; Figure 4). Postoperatively the patient had an elevation of amylase without deterioration of clinical signs. He gradually became asymptomatic and follow-up with sequential ultrasonograms showed resolution of the fluid collection. The pancreatic stent was removed a month later. At 3 months he was admitted for removal of the transgastric stents, but an abdominal X-ray showed that the stents had passed spontaneously. A new MRCP, at 6 months postinjury, showed complete disruption of the MPD with dilatation of the distal remnant (Figure 5). He remains asymptomatic, without steatorrhoea or diabetes, with normal amylase levels 7 months postinjury.

## Discussion

The pancreas, located in a relatively protected area of the abdominal cavity, is infrequently injured in typical blunt injuries. In a blunt trauma-induced pancreatic injury, fracture over the spinal column is usually observed. Isolated pancreatic injury can cause minimal symptoms early in the postinjury period and can be even silent in many cases [5,6]. The mechanism of injury should focus the clinician on the possibility of pancreatic injury.

Abdominal CT provides the best overall method for diagnosis and recognition of a pancreatic injury [7]. In cases in which the CT findings are inconclusive, further investigation with MRCP may be used. MRCP can demonstrate clear delineation of the MPD and its integrity [8].

Pancreatic injuries are classified into five grades according to the Pancreas Injury Scale published in 1990 by the American Association for the Surgery of Trauma [9]. Traditionally, patients with injuries to the MPD (grade III, IV, V) require laparotomy.

In our case a contusion and laceration of the pancreatic parenchyma was found on CT imaging. The integrity of the pancreatic duct could not be assessed by CT and MRCP was performed when the patient was transferred to our hospital, on the 6th day. He was haemodynamically stable with mild epigastric tenderness and, despite having a grade III injury, it was decided to treat it conservatively initially with the option of endoscopic intervention if required.

In general, injuries accompanied by MPD disruption require more aggressive treatments such as laparotomy or therapeutic endoscopy [10]. Huckfeldt *et al*. [11] reported the first successful stent placement when the procedure was performed a few hours after pancreatic trauma for MPD transection. Endoscopic transpapillary stenting of the MPD promotes healing of duct disruptions by blocking the leaking duct and bridging the disruption or by ablating the pancreatic sphincter converting the high-pressure pancreatic duct system to a low-pressure system with preferential flow to the duodenum [12,4]. Endoscopic transgastric drainage has been established for the treatment of peripancreatic fluid collections and pseudocysts after acute or chronic pancreatitis [13]. Therapeutic endoscopy has been used to treat duct disruptions in the early phase after pancreatic trauma or in the delayed phase to treat complications of duct injury [14,4]. However the published experience is limited.

Our patient had a full recovery and remains asymptomatic, but a duct stricture suggesting complete MPD disruption with upstream dilatation of the pancreatic duct has developed. According to the previous and recent imaging, ERCP to bridge the two MPD sections was considered impossible. Whether this can cause problems in the future is unknown.

## Conclusions

The clinical status of the patient rather than the grade of pancreatic injury should be the principal determinant to guide the diagnostic and therapeutic decisions. Endoscopic stenting and drainage is an attractive minimally invasive therapeutic procedure for haemodynamically stable patients, and it may obviate the need for surgery. However, worldwide experience is limited and further investigation is required regarding the safety and outcome.

## Consent

Written informed consent was obtained from the patient for publication of this case report and accompanying images. A copy of the written consent is available for review by the Editor-in-Chief of this journal.

## Abbreviations

CT: Computed tomography; ERCP: Endoscopic retrograde cholangiopancreatography; MPD: Main pancreatic duct; MRCP: Magnetic resonance cholangiopancreatography.

## Competing interests

The authors declare that they have no competing interests.

## Authors’ contributions

AV and VK designed the report; AV and GF were attending doctors for the patient; AV and AP performed the endoscopic procedure; GF and GP organized the report; AV and VK wrote the paper and GP and AP gave the final approval. All authors read and approved the final manuscript.

## References

[B1] Subramanian A Dente CJ Feliciano DV The management of pancreatic trauma in the modern era Surg Clin North Am 2007 87 6 1515 1532 10.1016/j.suc.2007.08.007 18053845

[B2] Jurkovich GJ Carrico CJ Pancreatic trauma Surg Clin North Am 1990 70 575 593 2190335 10.1016/s0039-6109(16)45131-5

[B3] Degiannis E Glapa M Loukogeorgakis SP Smith MD Management of pancreatic trauma Injury 2008 39 21 29 10.1016/j.injury.2007.07.005 17996869

[B4] Bhasin DK Surinder SR Rawal P Endoscopic retrograde pancreatography in pancreatic trauma: need to break the mental barrier J Gastroenterol Hepatol 2009 24 720 728 10.1111/j.1440-1746.2009.05809.x 19383077

[B5] Ahmed N Vernick JJ Pancreatic injury South Med J 2009 102 12 1253 1256 10.1097/SMJ.0b013e3181c0dfca 20016434

[B6] Bradley EL Young PR Chang MC Allen JE Baker CC Meredith W Reed L Thomason M Diagnosis and initial management of blunt pancreatic trauma Ann Surg 1998 227 6 861 869 10.1097/00000658-199806000-00009 9637549 PMC1191392

[B7] Phelan HA Velmahos GC Jurkovich GJ Friese RS Minei JP Menaker JA Philp A Evans HL Gunn ML Eastman AL Rowell SE Allison CE Barbosa RL Norwood SH Tabbara M Dente CJ Carrick MM Wall MJ Feeney J O'Neill PJ Srinivas G Brown CV Reifsnyder AC Hassan MO Albert S Pascual JL Strong M Moore FO Spain DA Purtill MA An evaluation of multidetector computed tomography in detecting pancreatic injury: results of a multicenter AAST study J Trauma 2009 66 641 646 10.1097/TA.0b013e3181991a0e 19276732

[B8] Gillams AR Kurzawinski T Lees WR Diagnosis of duct disruption and assessment of pancreatic leak with dynamic secretin-stimulated MR cholangiopancreatography Am J Roentgenol 2006 186 499 506 10.2214/AJR.04.1775 16423959

[B9] Moore EE Cogbill TH Malangoni MA Jurkovich GJ Champion HR Gennarelli TA McAninch JW Pachter HL Shackford SR Trafton PG Organ injury scaling, II: pancreas, duodenum, small bowel, colon and rectum J Trauma 1990 30 1427 1429 10.1097/00005373-199011000-00035 2231822

[B10] Lee PH Lee SK Kim GU Hong SK Kim JH Hyun YS Park do H Lee SS Seo DW Kim MH Outcomes of hemodynamically stable patients with pancreatic injury after blunt abdominal trauma Pancreatology 2012 12 487 492 10.1016/j.pan.2012.09.006 23217286

[B11] Huckfeldt R Agee C Nichols WK Barthel J Nonoperative treatment of traumatic pancreatic duct disruption using an endoscopically placed stent J Trauma 1996 41 143 144 10.1097/00005373-199607000-00024 8676408

[B12] Rogers SJ Cello JP Schecter WP Endoscopic retrograde cholangiopancreatography in patients with pancreatic trauma J Trauma 2010 68 3 538 544 10.1097/TA.0b013e3181b5db7a 20016385

[B13] Hookey LC Debroux S Delhaye M Arvanitakis M Le Moine D Deviere J Endoscopic drainage of pancreatic fluid collections in 116 patients: a comparison of aetiologies, drainage techniques, and outcomes Gastrointest Endosc 2006 63 4 635 643 10.1016/j.gie.2005.06.028 16564865

[B14] Coelho DE Ardengh JC Carbalo MT de Lina-Filho ER Baron TH Coelho JF Clinicopathologic characteristics and endoscopic treatment of post-traumatic pancreatic pseudocysts Pancreas 2011 40 3 469 473 10.1097/MPA.0b013e31820bf898 21343833

